# Effect of Aqueous *Allium cepa* and *Ixora brachiata* Root Extract on *Leishmania major* Promastigotes

**DOI:** 10.17795/jjnpp-15442

**Published:** 2014-04-24

**Authors:** Batool Sadeghi-Nejad, Jasem Saki

**Affiliations:** 1 Department of Medical Mycology, Cellular and Molecular Research Center, Ahvaz Jundishapur University of Medical Sciences, Ahvaz, IR Iran; 2 Department of Medical Parasitology, Infectious and Tropical Disease Research Center, School of Medicine, Ahvaz Jundishapur University of Medical Sciences, Ahvaz, IR Iran

**Keywords:** *Leishmania major*, Onions, Leishmaniasis

## Abstract

**Background:**

Leishmaniasis is a major worldwide public health problem with about two to three million humans threatened by this disease annually. *Allium cepa* (onion) is an important dietary vegetable and was used as a herbal medicine for centuries. The root of *Ixora brachiata* is medicinally important.

**Objectives:**

The aim of this study was to investigate the anti-Leishmania effect of the ethanolic and methanolic extracts of *Ixora brachiata* root and aqueous onion extracts on *Leishmania major* promastigotes.

**Patients and Methods:**

The parasites isolated from cutaneous leishmaniasis were exposed with different concentrations of selected plant extracts and their inhibitory effects on the promastigotes were evaluated after 24 and 48 hours.

**Results:**

Among tested plant extracts, *Ixora brachiata* root extracts revealed the best activity against *Leishmania major* promastigotes with IC_100_ value of 2.5 mg/mL and IC_50_ value of 0.078 mg/mL.

**Conclusions:**

This study showed that aqueous *Allium cepa* and *Ixora brachiata* root extracts as natural products could be used as alternative drugs in treatment of leishmaniasis.

## 1. Background

*Allium cepa* (onion), belongs to family Gallium, is rich in sulfur. It has been traditionally used as carminative, diuretic, expectorant, stomachic as well as antispasmodic, anthelmintic, and anti-infective factor (1). In addition to, *A. cepa* and its extracts have been used as a poultice for alleviation of skin diseases and insect bites (2). Antibacterial, antifungal, and anthelmintic activities of this vegetable and its preparations have also been reported (3). *Ixora brachiata* belongs to Rubiaceae family; it is a gigantic shrub or small tree that grows in rain forests. The root of *I. brachiata* is medically important and although its medicinal properties are not mentioned in medical texts, tribes are using it for skin diseases. Since review of literature revealed that leishmanicidal activity of the root of *I. brachiata* has not been evaluated so far, the present study was conducted to investigate the in-vitro antileishmanial activity of this plant. *Leishmania* is intracellular parasitic hemoflagellates that infect skin macrophages and leads to visceral leishmaniasis in vertebrate hosts. Despite cardiac and renal toxicity of pentavalent antimonies such as glucantime, these drugs are still the first choice among drugs used for the treatment of leishmaniasis (4). Due to side effects of commercial drugs for treatment of these diseases, it is necessary to find effective medicinal plants, easily available, and cheap drugs for prevention of the *Leishmania* growth. Traditional medicines are often a good source of bioactive compounds useful against many diseases such as leishmaniasis. Hence, we evaluated the antileishmanial effect of aqueous onion and *I. brachiata* extracts on the *Leishmania* promastigotes. Moreover, antileishmanial activity of many plant extracts were reported in previous studies (5-8). Although literature on *I. brachiata* root extract has shown good antidermatophytic activity (9), the effect of this plant has not been reported on *Leishmania* parasites up to this date. In addition, previous studies showed that all tested strains of *Leishmania* were sensitive to the onion juice (10).

## 2. Objectives

The aim of this study was to investigate the antileishmanial effect of the ethanolic and methanolic extracts of *I. brachiata* root and aqueous onion extracts against *Leishmania major* promastigotes.

## 3. Patients and Methods

### 3.1. Plant Material and Extract Preparation

The roots of *I. brachiata* were collected from district Ratnagiri, Maharashtra State, India. Fresh *A. cepa* (onion) was prepared from local market. They were flayed, cut into small pieces, and crushed in a blender. The homogenate was filtered through cleansing cloth. The filtrate was centrifuged and the supernatant of onion extract was filtrated, then sterilized with the help of 0.22 um Millipore filter (10) and stored at -20˚C. Aqueous *Allium cepa* extract was used immediately after the extraction. The dried and powdered leaves (10 g) from plants were extracted with ethanol 80 % by maceration. The crude extracts were obtained after evaporation under room temperature. According to procedure of described in previous studies, an aliquot of 1 g dried plant extracts was dissolved in 5 mL dimethyl sulfoxide (DMSO 1% solvent) as stock to prepare serial double dilutions to working concentrations of 0.078-20 mg/mL (11). All extracts were kept at 4˚C until used in future experiments.

### 3.2. Parasites

*The L. major* promastigotes were maintained in blood agar medium supplemented with RPMI-1640 medium (Sigma chemical Co.) and 5% fetal bovine serum (FBS) at 26˚C and were suspended in RPMI-1640 medium to adjust to a final concentration of 10 × 10^6^ parasites/mL.

### 3.3. Determination of the 50% Effective Concentration

The leishmanicidal activity of the plant extracts of aqueous *A. cepa* and *I. brachiata* root on parasites in the promastigote stage were assessed by using 96-well microplate. For determination of the 100% and 50% inhibition concentration (IC_100_ and IC_50_, respectively), each well was filled with 100 µL of the parasites suspension (1 × 10^6^ parasites/mL). Subsequently, 100 µL serial dilutions of the selected plant extracts were added to the same wells of microplate and the plate was incubated at 26˚C for 48 hours. A negative control (DMSO 1% solvent without any plant extract) and positive control (85 mg/mL of Glucantime) were used on the same plate. This is worthy of mention that neither the ethanol nor DMSO (up to 1%) used in tests had an effect on promastigotes (12). At the end of the incubation time, the plate was shaken over a plate shaker and the number of promastigotes in each concentration was calculated using a hemocytometer slide. Each assay was repeated three times. The surviving parasites were enumerated.

## 4. Results

**Figure 1.**
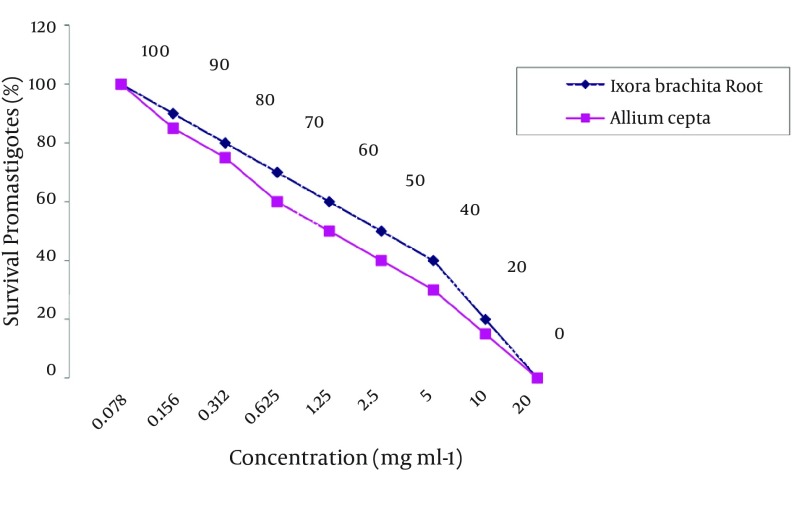
The Viability of *Leishmania major* Promastigotes in the Various Concentration of Aqueous *Allium cepa* and *I. brachita* Extracts

In the present investigation, extracts from selected plants were assayed for their in vitro leishmanicidal activity against *L. major* promastigotes cultures. The viability of *L. major* promastigotes in the concentration of 0.312 mg/mL ethanolic and methanolic extracts of *I. brachiata* root was 20% while over 80% of promastigotes in this concentration were passive. In addition, at the concentration of 2.5 mg/mL ethanolic and methanolic extracts of *I. brachiata* root, 100% of the *L. major* promastigotes were unmovable and the viability of the *L. major* promastigotes in the same concentration was 0%. The viability of the *L. major* promastigotes in the concentration of 0.312 mg/mL aqueous onion extracts was 80% and in the same concentration, 20% of the *L. major* promastigotes were unmovable. At the concentration of 2.5 mg/mL aqueous *A. cepa* extracts, 70% of the *L. major* promastigotes were unmovable and the viability of promastigotes was 30%. Moreover, in the concentration of 5.0 mg/mL of aqueous *A. cepa* extracts, 100% of the *L. major* promastigotes were unmovable and the viability of parasites in this concentration was 0% (Table 1 and Figure 1).

**Table 1.** In Vitro Antileishmanial Activity of *Ixora brachiata* Root and Aqueous *Allium cepa* Extracts ^a^

Plant Material	Promastigotes	
	IC_100 _Value, mg/mL	IC_50_ Value, mg/mL
**Ethanolic extract of ** ***Ixorabrachiata*** ** root**	2.5	0.078
**Aqueous onion**	5.0	1.25
**Control drug**		
Glucantime	8.5	21.25

^a^ IC_50_ and IC_100_ are the sample concentrations that inhibited 50% and 100% the growth of parasites.

## 5. Discussion

Saleheen et al. (10), have reported aqueous *A. cepa* extracts (AOE) gave an IC_100_ and average IC_50_ values of 1.25 mg/mL and 0. 376 mg/mL, respectively, against tested *L. major* promastigotes (10). These figures are close to our finding in the present study. According to the mentioned reports above, the *L. major* promastigotes inhibited by the ethanolic and methanolic extracts of *I. brachiata* root had low viability (20%) while the *L. major* promastigotes inhibited with aqueous *A. cepa* extracts had high viability (80%). It was interesting to note that the ethanolic as well as methanolic extracts of *I. brachiata* root and aqueous onion revealed potential leishmanicidal activity, as shown in Tabe1, with the IC_50_ values of 0.078 and 1.25 mg/mL, respectively. These were comparable to the effects of glucantime (IC_50_ = 21.25 mg/mL) used as positive control. The IC_100_ values of tested plant extracts were 2.5-5.0 mg/mL, which were comparable to the IC_100_ of 85 mg/mL glucantime. These results revealed that the the tested plant extracts had high potential antileishmanial activity in comparison to glucantime (IC_50_ of 21.25 mg/mL and IC_100_ of 85 mg/mL). However, according to the previous studies, some other plant extracts such as *Allium sativum*, *Plagiochila disticha,* and *Casearia sylvestris* also have exhibited inhibitory activities against *Leishmania* (13). Moreover, the presence of compounds such as tannins, flavonoids, saponin carbohydrates, coumarins, and triterpenes reported by previous studies (9), may be responsible for leishmanicidal activity in this plant. These results are supported with the observations of Marine et al. and Firdous et al. (14, 15), who have shown the leishmanicidal activity of the flavonoids isolated from *Consolida oliveriana* and the efficacy of the carbohydrates in treatment of leishmaniasis. In addition, tannins isolated from *Anogeissus* *leiocarpus* was shown to possess a good activity against *Leishmania* (16). Other compound such as terpenoids (sesquiterpene lactones) have been reported to be active against *Leishmania* (17).

This study revealed that selected plants in this investigation contained potent compound with high potential leishmanicidal activity. Although glucantime is very toxic, it is still used because other drugs have been shown to be of variable effect or ineffective against the parasites (18). Since commercial drugs for treatment of leishmaniasis have many side effects, it would be timely to find effective medicinal plants in herbal medicine for the investigation and isolation of their active compound and the study of their toxicity.
